# Laser-Induced Microgrooves Improve the Mechanical Responses of Cemented Implant Systems

**DOI:** 10.3390/mi11050466

**Published:** 2020-04-29

**Authors:** Morshed Khandaker, Abdellah Ait Moussa, Desmond Nuyebga Sama, Fereshteh Safavinia, Susmita Hazra, Onur Can Kalay, Fatih Karpat, Erik Clary, Amgad Haleem

**Affiliations:** 1 Department of Engineering and Physics, University of Central Oklahoma, Edmond, OK 73034, USA; 2 Department of Physics, Cameron University, Lawton, OK 73505, USA; 3 Department of Mechanical Engineering, Bursa Uludag University, Bursa 16059, Turkey; 4 Department of Veterinary Clinical Sciences, Oklahoma State University, Stillwater, OK 74078, USA; 5 Department of Orthopedic Surgery, University of Oklahoma Health Science Center, Oklahoma City, OK 73104, USA; 6 Department of Orthopedic Surgery, Cairo University College of Medicine, Cairo 11562, Egypt

**Keywords:** bone cement, digital image correlation, laser grooving, implant–cement interface, total joint replacement

## Abstract

The impact of a laser-induced microgroove (LIM) architecture on mechanical responses of two cemented implant systems was evaluated. One system consisted of two aluminum alloy rods bonded end-to-end by polymethylmethacrylate cement. The second system consisted of a custom-made, aluminum tibial tray (TT) cemented in an artificial canine tibia. Control specimens for each system were polished smooth at the cement interface. For LIM samples in the rod system, microgrooves were engraved (100 µm depth, 200 µm width, 500 µm spacing) on the apposing surface of one of the two rods. For TT system testing, LIM engraving (100 µm spacing) was confined to the underside and keel of the tray. Morphological analysis of processed implant surfaces revealed success in laser microgrooving procedures. For cemented rods tested under static tension, load to failure was greater for LIM samples (279.0 ± 14.9 N vs. 126.5 ± 4.5 N). Neither non-grooved nor grooved TT samples failed under cyclic compression testing (100,000 cycles at 1 Hz). Compared with control specimens, LIM TT constructs exhibited higher load to failure under static compression and higher strain at the bone interface under cyclic compression. Laser-induced microgrooving has the potential to improve the performance of cemented orthopedic implants.

## 1. Introduction

Total joint replacement (TJR) is a surgical procedure in which the surfaces of a diseased joint are excised and replaced with two metal prostheses and a polyethylene articulation between them. To secure the prostheses in place, TJR systems may employ either bone cement (polymethylmethacrylate (PMMA)) or a press-fit strategy. The cemented approach is generally reserved for osteoporotic bone whereas cementless fixation requires healthy bone for immediate (via press-fit) and longer-term (via bony ingrowth) stability [1,2]. An ideal implant for TJR surgery should deliver lifelong stability within the adjacent tissue [3]. If the bone–implant interface proves inadequate, micromotion will occur and lead to activation of cells (osteoclasts) that resorb bone at the interface, further exacerbating implant loosening and leading to eventual failure [4]. In the U.S. alone, approximately 40,000 hip arthroplasty surgeries have to be revised each year, and the rate is expected to increase by 137% (and by 601% for total knee revisions) over the next 25 years as the population ages [5]. Improving TJR implant durability, therefore, is of the utmost clinical importance [6]. 

The weight-bearing capacity of joint replacement devices is critical to their success, but in transferring load away from the surrounding bone, they may elicit bone resorption that, if severe enough, can lead to implant loosening and even catastrophic fracture of the bone [7]. This phenomenon of “stress shielding” is well recognized and has prompted much research aiming for implant systems that deliver immediate stability, promote early osseointegration, and are customizable to match patient build, physical condition, and level of activity.

Another critical factor relating to the potential for implant loosening of cemented TJR is the mechanical behavior at the implant–cement interface. Recent research suggests that a rough implant surface may enhance cemented bonding and thereby increase immediate shear-load bearing capacity of the bone–implant construct [8]. Recent advancements in the field of metallurgy are yielding other strategies for improving implant stability by altering TJR implant surface morphology in a way that improves the immediate stability and promotes the transfer of the weight-bearing force to the bone envelope [9]. One potential strategy is to increase the surface area of the implant’s cement interface. 

In previous research, we etched microgrooves in metal implants using a precision diamond sawing machine [10]. Our preliminary studies showed that microgrooving cementless titanium (Ti) implants significantly improve biocompatibility, mechanical stability, and osseointegration of the device [11,12]. Laser-induced micro- and nanotexturing that increase the surface area and roughness may further enhance the stability of press-fit implants by increasing friction [13]. To date, no study has reported the effect of microgrooving on the mechanical performance of cemented TJR implants. In the present publication, we report our evaluation of a novel laser-induced microgroove (LIM) architecture on the mechanical performance of two cemented aluminum alloy devices—one a bonded rod system and the other a total knee replacement (TKR) tibial tray—working on the hypothesis that LIM would enhance cement bonding and improve mechanical performance. 

## 2. Materials and Methods 

### 2.1. Materials

Aluminum (alloy 6061) was used to manufacture implants (rod and tibial tray (TT)) and fixtures for the mechanical tests. An artificial canine tibiae (SAWBONES^®^, Vashon Island, Washington, USA, SKU # 2117-33) were used in TT assessments. This model bone is made of polyurethane foam material and features a rigid outer “cortical” shell and a spongy “cancellous” inner core that replicate normal canine bone. This model is amenable to easier cutting and drilling compared to the typical 3D-printed plastic cortical shell models. 

### 2.2. Sample Design 

For this study, straight rods and tibial tray implants were designed and manufactured with three smooth (“control”) and three LIM (“test”) samples for each device. Surface morphology was evaluated on one sample randomly selected from each group. Each cemented rod implant system consisted of two rods. The adjoining flat surfaces of both rods were polished smooth for control specimens, whereas test samples featured one rod microgrooved on the end after polishing. Tension tests under static loading were performed after bonding each sample’s rods with PMMA cement. For each tibial tray, the underside and keel of the tray (bone interface) were polished and microgrooving then performed only on the test samples. Compression tests were conducted on each sample under a static loading condition (*n* = 1) to measure the load vs. deformation characteristics that were then used to determine the preload and applied load for compression tests under cyclic loading. Cyclic tests were conducted on each sample group (*n* = 2) to measure the strain transfer from the TT to the adjacent bone for a specific cycle interval and test for failure up to 100,000 cycles. A finite element analysis model was developed from the cemented TT system to evaluate the strain transfer from the implant to the bone.

### 2.3. Sample Preparation

#### 2.3.1. Cemented Rod Implants

A diamond saw machine sectioned twelve 50 mm-long segments from a long aluminum rod of diameter 12.7 mm. One end of each rod was polished using grinding paper up to 1500 grit, ultrasonically rinsed in acetone, ethanol, and deionized water for 15 min each, and then air dried. A metal laser engraving system (Full Spectrum Laser, Galvo FP, Las Vegas, NV, USA) machined a set of parallel microgrooves (100 µm depth, 200 µm width, 500 µm spacing) on the polished end of three segments. For each rod pair, one rod was placed in the upper grip and the other rod in the lower grip of a universal testing machine (Test Resources) with polished ends facing each other. 

#### 2.3.2. Cemented Tibial Tray Implant

##### Geometry and Modeling

**Figure 1:**
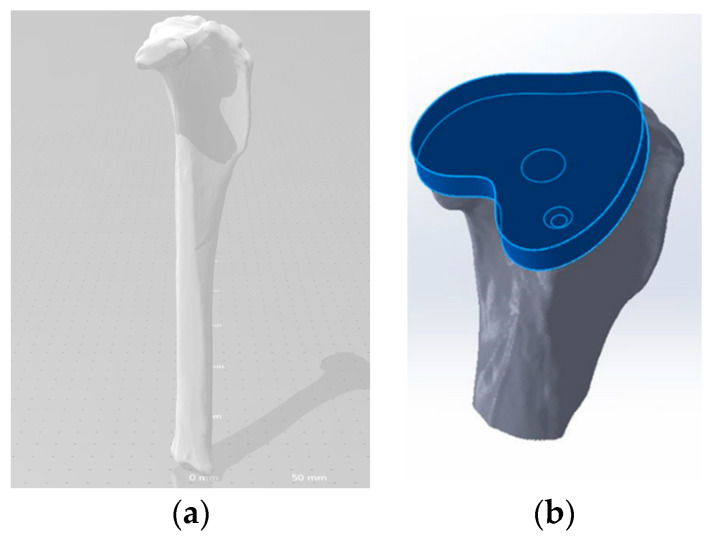
Process to model a tibial tray (TT) from a canine tibia bone. (**a**) CT scan model of a SAWBONES canine tibia and (**b**) Geometric modeling of a tibial tray from a proximal tibia end profile.

One artificial canine tibia was scanned using a multi-laser scanner (ProImage 3D, Tulsa, Oklahoma, OK, USA). A 3-dimensional model of the bone was rendered in Drawing Exchange Format (.dxf) format and imported into MeshLab to convert the file into an Initial Graphics Exchange Specification (.iges) file type for subsequent importing into SolidWorks^®^ (Dassault Systemes, Waltham, MA, USA) software (Figure 1a). Next, a three-dimensional solid model of a tibia tray implant matching the profile of the proximal tibial TKR bone cut was generated (Figure 1b). 

##### Tibia Tray Manufacture

**Figure 2:**
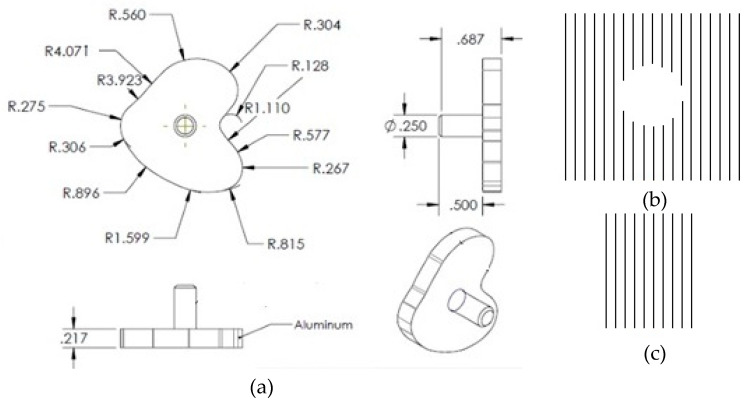
(**a**) Bottom, front, side, and isometric views of a tibial tray implant which was used to manufacture the TT implant. Schematic representation of diagrams used to produce laser patterning on the (**b**) bottom flat and (**c**) keel surface. The gap between two lines was 300 µm.

The keel for the tibial tray was modeled as seen in Figure 2a following the keel geometry of David et al. [14] without any peripheral extension. Using the solid model, a computer numerical control (CNC) machine was used to fabricate all tibia trays from a 0.75″ thick × 6″ wide, 6″ long 6061 aluminum bar (Mcmaster Carr, Los Angeles, CA, USA). Each tray was polished using grinding paper up to 1500 grit, ultrasonically rinsed in acetone, ethanol, and deionized water for 15 min each, and air dried. For test samples, microgrooves (100 µm depth, 200 µm width, 100 µm spacing) were laser engraved on the underside of the tray and entire circumference of the keel (Figure 2b,c). Laser-grooved samples were ultrasonically rinsed in acetone, ethanol, and deionized water for 15 min each and then air dried.

##### Cemented Tibia Tray/Bone Sample Preparation

**Figure 3:**
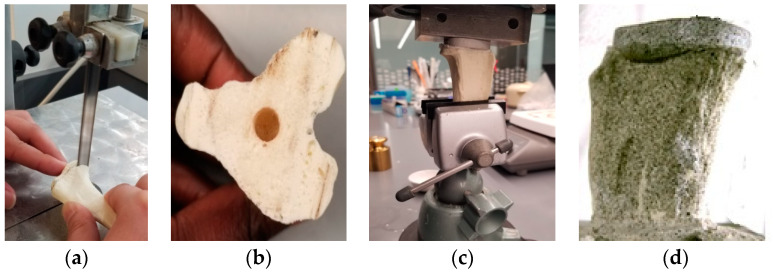
Process to create experimental TT–bone samples. (**a**) Proximal tibia cut using a butcher saw, (**b**) Drilled TT keel hole to secure tibial tray using bone cement, (**c**) Maintenance of alignment during the curing of a tibial tray in bone using bone cement, (**d**) a white and black spray-painted TT–bone sample that is used for mechanical tests.

Transverse cuts were made in the proximal end of the six canine SAWBONES following a TKR osteotomy protocol (Figure 3a). A laboratory retort stand with two utility clamps was used to secure the bone model. The bottom clamp was positioned 25 mm above the distal end of the bone and the top clamp 7 mm below the proximal end. Clamp position was adjusted such that the axis of the stand was parallel to the center (mid-sagittal) axis of tibia, and the two clamp shafts were parallel. The proposed cut was marked by pencil, and a butcher saw was used to cut along the marked line. The center axis of the tibia was marked on the cut surface, and a hole was drilled into the bone at that point using a ^3^/_8_ inch drill bit to a depth of 0.5 inches (Figure 3b). Biomedtrix^®^ PMMA bone cement was used (2.4 g PMMA bead with 1.2 mL of MMA monomer). The cement mixture was stirred for 30–40 s to obtain a homogenous mixture and poured into the drilled hole. The keel of the tibial tray was pressed into the hole and after establishing the proper rotational alignment, a set of weights equivalent to 7.5 kg was placed on top of the tray TT until polymerization was complete (approximately 30 min; Figure 3c). The particular weight distributed over the total surface area of the tray (1.28 × 10^−3^ m^2)^ replicated a curing pressure of 60 kPa that is employed clinically [15]. To facilitate subsequent measuring of the strain around the bone–implant interface, white and black spray paint was applied on the surface of the sample. A coat of white paint was applied to the whole surface of the sample, and then the random black speckles were applied (Figure 3d).

### 2.4. Experiments and Analysis

#### 2.4.1. Topographical Characteristics 

A three-dimensional, optical profilometer (Filmetrics, San Diego, CA, USA) was used to analyze the roughness of the treated surfaces of one randomly selected specimen from control and test groups for each device (rods and tibial trays). From the profilometer data, the arithmetic mean deviation (*R*_a_) was calculated. The scanned profilometer image was also used to measure microgroove dimensions in the grooved sample. Profilm3D consists of white light interferometry (WLI), which is used to measure surface profiles and roughness down to 0.05 µm. It also includes the phase-shifting interferometry (PSI) mode option, which measures the minimum vertical estimate size down to 0.001 µm, which was used to measure the depth of the microgrooves created by laser micromachining.

#### 2.4.2. Mechanical Characteristics

##### Cemented Rod Implants under Static Tension

**Figure 4:**
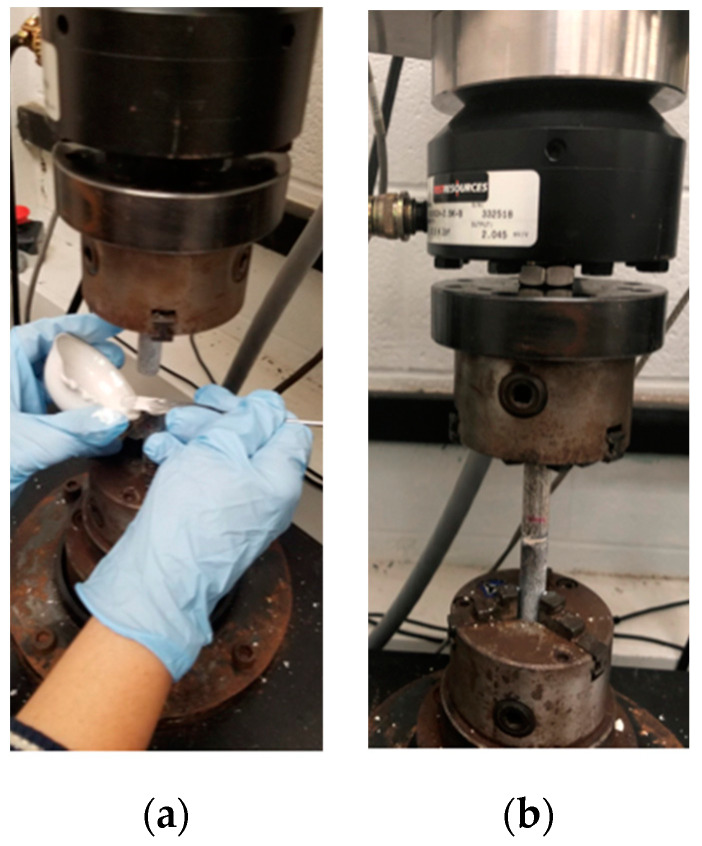
Preparation of cemented rod samples in UTM: (**a**) cement pouring on top of the bottom rod and (**b**) a cemented rod sample pointing to gaps filled by cement.

Biomedtrix^®^ PMMA bone cement (Whippany, New Jersey, USA) was used (2.4 g PMMA bead and 1.2 mL of MMA monomer) to bond rod surfaces. The adjoining flat surfaces of each rod were smooth in the control group (*n* = 3), whereas the top surfaces of the bottom rod were laser engraved for test samples (*n* = 3). The mixture was stirred for 30–40 s to obtain a homogenous mixture. Bone cement paste was poured on the top of the bottom rod sample (Figure 4a). The top gripper was lowered such that there was a gap of 2 mm between the two pieces, and the deposited PMMA cement was compressed into the gap (Figure 4b). Excess cement around the interface was cleaned. The cement was cured for 30 min before a tension test. Tension tests were performed on the smooth and grooved rods until failure of the implant/bone interface. 

##### Cemented Tibial Tray Implants with Bone

A. Static Compression

**Figure 5:**
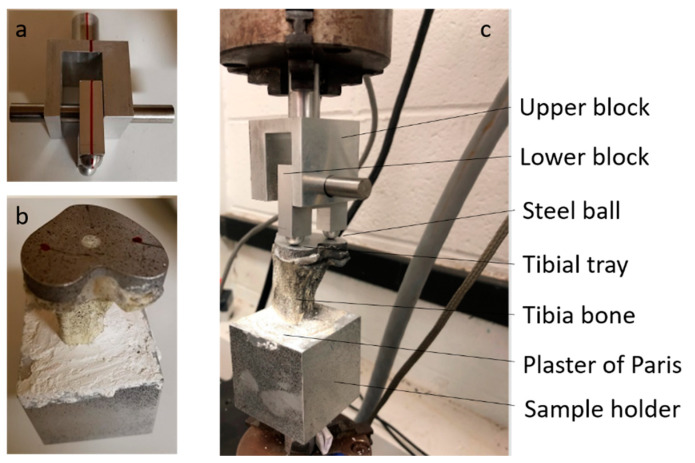
Preparation of cemented TT samples in UTM: (**a**) mechanism attached with UTM top gripper. The red line shows alignment of balls with the upper block center axis, (**b**) cemented TT samples. Red dots indicate indentation positions, (**c**) mechanical tests on a cemented TT sample.

A custom-made sample holder was manufactured to secure the tibial tray to the bottom gripper of the UTM. The holder was filled with plaster of Paris, and the sample was placed at the center of the holder. A support stand hardware equipped with an extension clamp was used to maintain the vertical position of the sample during the overnight curing of the plaster of Paris. A custom-made mechanism (Figure 5a) [16] attached to the top gripper applied compressive load to the tray. The mechanism consisted of two blocks. The upper block, indexed to the tibial tray, supported the lower block via a pin mechanism. The upper block was secured with the top gripper of the UTM, whereas the lower block had two arms with ball ends. The shaft of the upper block was concentric with the center axis of the tibia and TT keel (Figure 5b), for which the two balls equally distributed the compressive load from the UTM crosshead to the lateral and medial sides of the tray. This mechanism provided anterior–posterior (AP) translation and internal–external (IE) rotation during the compression tests that represent the contribution of the TT implants to passive joint constraint following TKR surgery [17]. Compression tests were performed under static (freestanding) loading conditions on the smooth and grooved TT (*n* = 1) by increasing the displacement at a rate of 0.05 mm/second until failure of the implant/bone interface. The UTM automatically recorded the force and displacement. The compression test ended when a 100% drop of force occurred.

B. Cyclic Compression

B.1. Failure Analysis

Compression tests were performed under a cyclic loading condition on the smooth (*n* = 2) and grooved (*n* = 2) TT samples. A zero load and then sine waveform load with mean of 150 N and amplitude of 30 N were applied to each sample. This value was calculated as three times the bodyweight (45 lb) of a typical medium-sized dog distributed over four legs (200 N). A total of 100,000 cycles at 1 Hz was applied to mimic the load and displacement behavior under cyclic (running or walking) conditions [18]. Every 1000 cycles, the displacement of the specimen was recorded from the UTM. A high-speed camera (Phantom V641) captured images along with the TT–bone interface at no load and at the 11,000th and 27,000th cycles. 

B.2. Micromotion Analysis

B.2.1. Digital Image Correlation (DIC) Method

An open-source MATLAB (MathWoks Inc, Natick, MA, USA) DIC algorithm was used for calculating the strain along the TT–bone interface working from the high-speed camera data obtained during the cyclic compression tests [19]. The DIC algorithm created a grid on an image of a pre-loaded specimen and treated the picture as a reference image for successively loaded samples during the cyclic test. The x-position and y-position of pixels in each image were recorded. The subset size was chosen from 10 × 10 to 100 × 100 (size of the grid in pixel) that determined the resolution of grids. In the speckled surface paint pattern, the subset size was one of the essential parameters in the accuracy of the results in the DIC method. Different subset size checks for each experiment were performed to find the best resolution depending on the speckle pattern. The program found correlation based on displacement data of a series of images before and after deformation. Strain along the TT–bone interface at different cycles during the fatigue tests was measured by a successive comparison of the x-position and y-position from the captured images at corresponding periods using the DIC algorithm. 

B.2.2. Finite Element Method

A computational finite-element model for strain in the TT constructs was developed using ANSYS 14.5 FEA software (ANSYS Inc, Canonsburg, PA, USA). SolidWorks software (SolidWorks^®^ from Dassault Systems, Waltham, MA, USA) was used to generate models of both smooth and grooved trays (Figure 2). A fatigue analysis was then performed to compare the strain between the experimental and computational models at the TT–bone interface. 

SolidWorks^®^ software was used for the proximal tibial cut, making a hole for cementing the keel and assembling the TT at the center of keel hole according to the experimental sample. The cement thickness was considered to be 2.5 mm between the TT and the bone [14]. The bone, cement, and the tibial tray models were assumed to be homogeneous, linear elastic, and isotropic. The material properties of the assembled design used in this study are presented in Table 1. 

**Figure 6:**
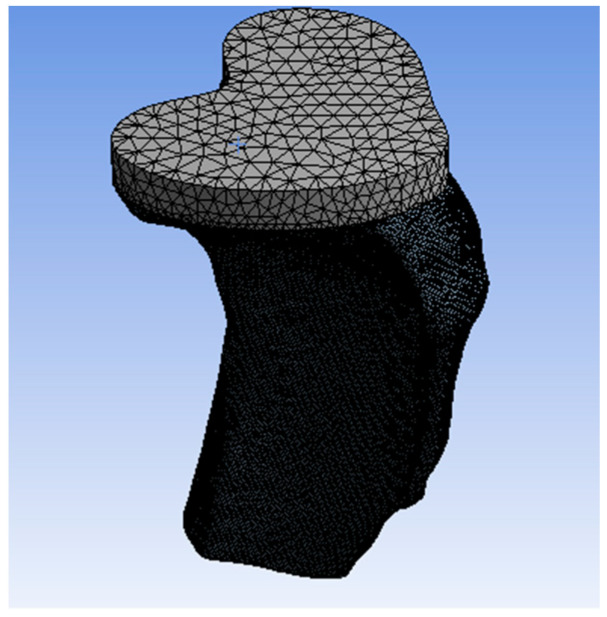
Mesh structure of the FEA model.

Figure 6 shows the meshed structure of the study model. The tetrahedral mesh structure was preferred for the bone model given the complex anatomy of the bone [22]. The mesh size was defined as 1 mm. For high-quality results, the element size was improved for the implant–bone interface and was defined as 0.5 mm. The same procedure was followed for both the non-grooved and the grooved models. The mesh structure for the non-grooved model had 135,324 elements and 221,093 nodes, whereas the mesh structure for the grooved model had 166,060 elements and 271,658 nodes.

The contact between the implant–cement and the cement–bone was modeled with the node-to-surface algorithm. The interfaces between the cement and implant were simulated with a friction coefficient of 0.25 and 0.30 for the implant/bone interface [22]. The friction coefficient for cement–bone was defined as 0.3, according to Janssen et al. [23]. To avoid unnecessary CPU usage, the 3D model of the tibia was truncated halfway through its longest dimension (Figure 6), and the fixed support was applied from the distal end of the tibia [20]. The balls were modeled with the point contact in FE simulations to represent cyclic compression test conditions. A mean load of amount 75 N and amplitude 15 N was applied from each ball, as a total load of 150 N from the UTM was equally distributed across two balls in our experimental model. The outcome of the finite element analysis for both non-grooved and grooved models was the equivalent to von Mises strain values along with the TT–bone interfaces. The results were compared with the experimental results to determine the effect of LIM on TT related to the mechanical stimuli distributions from the implant to the bone.

#### 2.4.3. Statistical Analysis

Numerical data are presented as the mean ± standard deviation. In analyzing mean values between smooth and grooved groups, independent samples t-tests were performed with the assumption of unequal variances. For all statistical tests, *p* < 0.05 was considered as the statistical significant comparison.

## 3. Results

### 3.1. Topographyical Chracteristics

**Figure 7:**
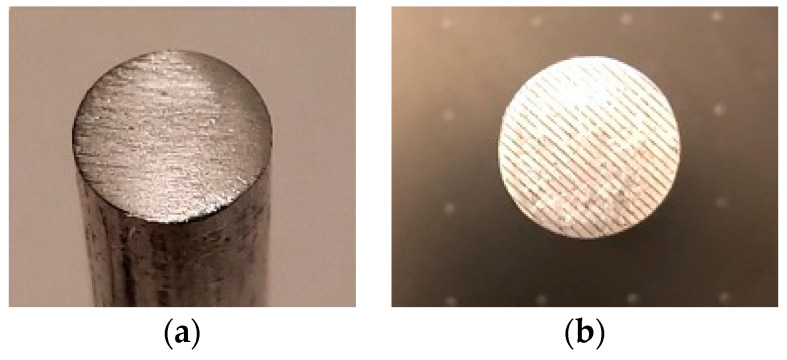
Manufactured rod samples: (**a**) smooth and (**b**) grooved.

**Figure 8:**
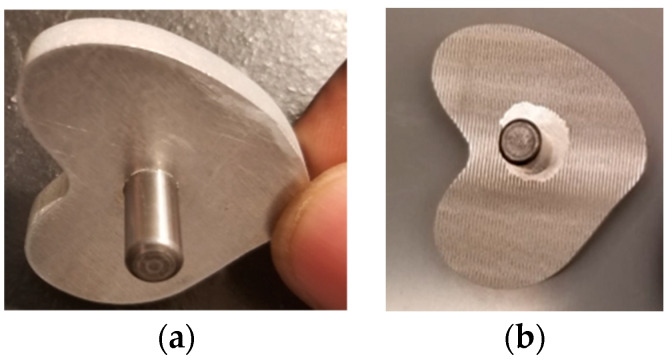
Manufactured TT samples: (**a**) smooth and (**b**) grooved.

This study manufactured three smooth and three grooved rod and TT samples. Figure 7 and Figure 8 show randomly selected smooth and grooved samples. Laser-induced microgrooves fully covered rod flat surfaces (Figure 7b). Only near the connection of the keel to the tray was microgrooving (Figure 8b) challenged (due to the misalignment of the laser head center axis with the center axis of the keel in TT). 

**Figure 9:**
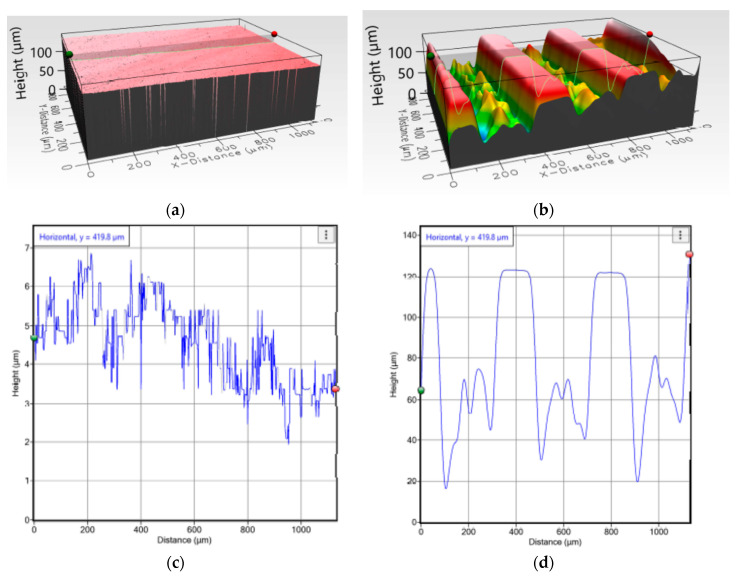
3D topographical view of (**a**) smooth and (**b**) grooved TT samples. Roughness profiles along an arbitrary line on a (**c**) smooth and (**d**) grooved samples were determined from Figure 9a,b, respectively.

A clear topographical difference of smooth (Figure 9a) and grooved (Figure 9b) TT was observed. Uniformly spaced microgroove profiles were visible from the images, whereas no significant roughness was found in smooth samples (maximum peak height = 6.568 µm). The profiler image showed that the shape of the groove was trapezoidal. The approximate space between adjacent microgrooves (depth = 100 µm and width = 200 µm) was 100 µm. The topography showed significant roughness at the trough of the microgrooves. The calculated roughness for grooved trays was significantly higher (3.62 ± 0.08 µm, *n* = 5) than that for smooth samples (1.74 ± 0.08 µm, *n* = 5) (*p* < 0.05). 

### 3.2. Mechanical Chracteristics

#### 3.2.1. Cemented Rod Implants

LIM improved cement bonding of the aluminum rods. Under static tension, mean force to failure was significantly higher for grooved rod constructs (279.0 ± 14.9 N, *n* = 3 vs. 126.5 ± 4.5 N, *n* = 2 for smooth constructs). Excluded from the analysis was one control sample that failed at a very low force (4.3 N).

#### 3.2.2. Cemented TT Implant with Bone

##### Load vs. Displacement Characteristics

**Figure 10:**
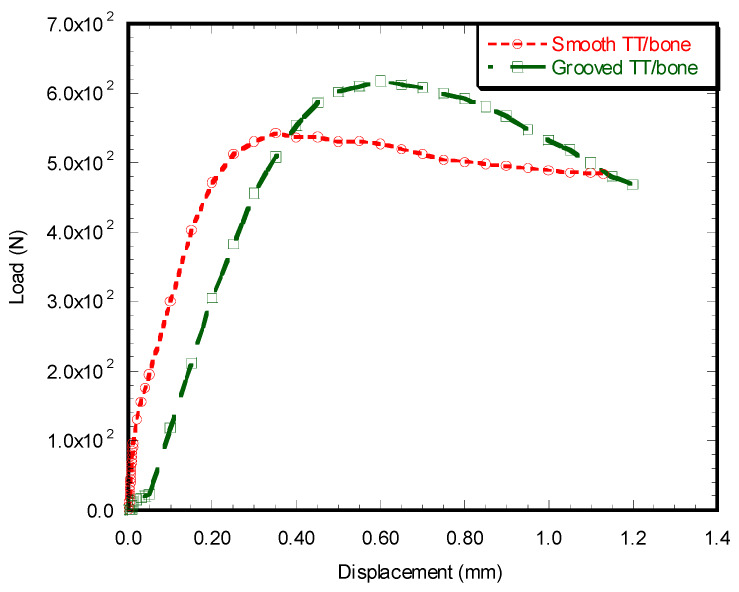
Load vs. displacement plot of smooth TT–bone and grooved TT–bone samples under compression load.

Figure 10 shows the observed load vs. displacement characteristics of a smooth and grooved sample during the static compression tests. A higher initially elastic response, broader inelastic region, and smoother descending load–displacement response were observed for the grooved samples compared to the smooth samples. Figure 10 depicts a higher amount of force and displacement for the failure of a grooved sample in comparison to a smooth sample.

**Figure 11:**
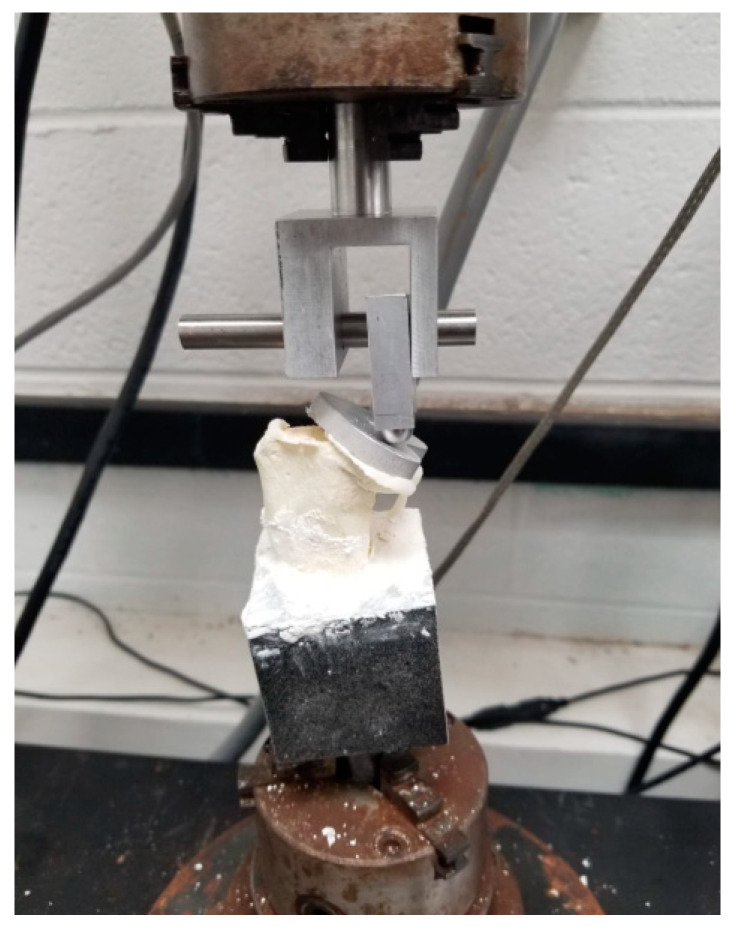
A collapsed sample from the static compression tests. A significant deformation occurred before the failure of the samples.

There was a significant amount of deformation after reaching the maximum peak force for both smooth and grooved samples until the detachment of the implant from the bone surface. Figure 11 shows a tray detached from the bone sample at the end of the experiment. For both smooth and grooved samples, separation occurred at the anterior aspect of the tray along the cement–bone interfaces. With the smooth TT–bone sample, there was no remaining attachment of bone to cement at failure, whereas a significant attachment of bone to cement was observed in the grooved TT–bone sample. 

Fatigue test results showed no failure of smooth TT–bone (*n* = 2) and grooved TT–bone (*n* = 2) interfaces after 100,000 cycles. There was no change in sample height after 100,000 cycles for both samples due to the applied cyclic load. This means that although there might be internal deformation in bone, there was no permanent strain on each sample upon unloading of the sample. Additionally, visual inspection of the TT–bone interface before and after the experiment found no separation of the interface after the test. 

##### Microstrain Analysis around the TT–bone Interface

DIC analysis of captured images after 11,000 and 27,000 cycles revealed higher strain values along the TT–bone interface for LIM grooved samples compared to the strain values of smooth samples (Table 2). 

**Figure 12:**
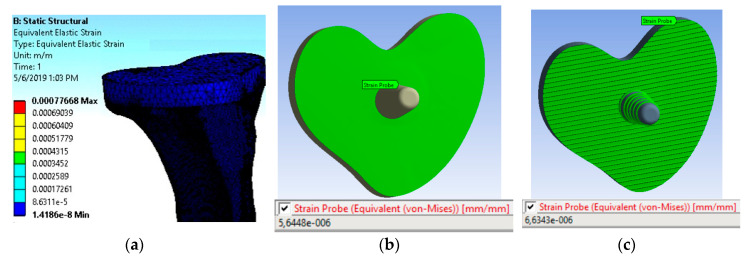
Computation analysis of the TT–bone model after 11,000 cycles. (**a**) Equivalent Von Mises strain contour plot of the entire TT–bone sample. Von Mises strain along the TT–bone interface on (**b**) smooth and (**c**) grooved samples.

Finite element analysis (FEA) of smooth and grooved models after 11,000 cycles showed increased strain values for grooved samples compared to smooth samples along the TT–bone interface (Figure 12). FEA reported the average strain distribution along the TT–bone interface, which was highlighted in green for smooth (Figure 12b) and grooved (Figure 12c) samples since the variation in strain distribution from TT to the tibia bone could not be seen from the entire body image (Figure 12) via ANSYS© “strain probe.” The difference in the average microstrain between the smooth and grooved samples from DIC analysis was 36%, whereas the difference in the microstrain from the FEA model between the sample groups was 17.85%. Although the TT–bone interface bonding conditions between experimental and finite element models for smooth and grooved samples were different due to laser machining limitation, both DIC and FEA found increased strain values at the TT–bone interface due to microgrooves on TT.

## 4. Discussion

In this study, the novel micromachining process proved accurate and reliable in microgrooving the surface of aluminum alloy implants. Laser-induced microgrooving improved the bonding between the implant and cement by enhancing the interlocking of cement via increased roughness and larger surface area of the implant. The effect of improved interlocking on failure can be easily seen from the static tension test results. LIM created mechanical interlocks that resulted in higher cement attachment to the implant surface. This created higher bonding between the cement and rod that resulted in higher maximum tension force for grooved cemented rod samples compared to smooth cemented rod samples. Additionally, the scheme increased the amount of transfer of load from the implant to the bone. Topography and mechanical test results suggested that there exists a correlation between implant surface roughness and levels of microstrain along the implant–cement or cement–bone interfaces. Furthermore, laser-induced micromachining of a TKR implant may control the amount of micromotion from the implant to the bone by regulating the amount of implant surface roughness and solving the implant loosening due to stress-shielding effects. 

The amount of implant-to-cement contact area determines the mechanical stability of implants [3,7]. Therefore, the effect of laser-induced microgrooves on strains at the implant–cement interfaces was examined using experimental and computational models. The experimental results found a higher level of mechanical stability for the grooved rod and tibial tray implants in comparison to the smooth implants due to the larger implant to cement contact surface area resulting from LIM (Figure 10). Because LIM contributes to greater interlock between the implant and cement on grooved implants, higher elastic strain energy was found in grooved TT–bone samples than in smooth TT–bone samples. We also observed higher attachment of bone for grooved TT–bone samples compared to smooth TT–bone samples. This can be explained by the fact that LIM created mechanical interlocks that resulted in higher transfer of load from the cement to the bone. This created greater bonding between the cement and bone in the case of grooved/TT samples compared to smooth TT–bone sample. Our study suggests that implant surface roughness greatly impacts the overall mechanical properties at the implant–cement interface and so aligns with other researcher findings. Walsh et al. [24] investigated the influence of surface roughness using polymethylmethcrylate (PMMA) and a bisphenol-a-glycidylmethacyrlate resin-hydroxyapatite cement (CAP). Their study found that increasing surface roughness improved the mechanical properties at the implant–cement interface for both types of cement. Similarly, in a previous study, we showed that circular shape nano-roughness of titanium implants created by controlled laser peen treatment improved the union between titanium–cement interfaces [25]. 

The cement to bone contact in TT–bone samples redistributes applied force to the adjoining bone. The flexion and extension over the anterior region of the tibial tray due to cyclic compression load produced both normal and shear strain along the cement–bone interface in vitro and in vivo. The resultant strain may likely have caused the cement–bone interface failure (Figure 11). The increase of the surface area of the TT surface using laser micromachining showed a positive influence of increasing microstrain at the adjoining bone, therefore LIM on the TT surface can be considered a factor for mechanical stimuli from the implant to the bone. A future study goal is to find a specific LIM topography for an implant that may provide sufficient mechanical stimuli from the implant to the bone to eliminate stress-shielding effects. Our developed FEA model can be used to achieve this goal by determining the relationship between microgroove architecture and stress-shielding factors [26]. Since laser micromachining techniques can produce the specific microgroove architecture, our FEA model can assist in designing stress shielding-free total joint replacement implants.

LIM surface architecture has potential implications in improving osseous integration in both cemented and cementless joint replacement implants. Surface topography of implants is known to influence the rate of bone remodeling surrounding the implant [27,28]. Other researchers have found strong influences of groove topography on the stress transfer from implants to bones for initiating the bone remodeling process [27,28]. Therefore, LIM can have a positive influence on initiating the bone remodeling process in a cemented joint replacement implant. Previous studies have identified micron to nanoscale surface osseointegrated features of implants, such as microroughness [29], microporosity [30], and nano-roughness [31] as the potent modulators of cellular functions through the onset of focal adhesion formation. Microscale topographies can be created on cementless implant systems using LIM, allowing an extracellular matrix (ECM) to be deposited along the groove, enhancing the mechanical stability and osseointegration of the implant with the host tissue.

A tibial tray in a currently available TKR system includes multiple peripheral extensions that provide mechanical stability of the TT against torsional rotation. This study was limited to evaluate the effect of LIM on strain distribution due to uniaxial load (tension and compression) only, and our current LIM schemes cannot adequately treat these peripheral extensions. Therefore, our TT geometric model did not include any outer extensions from the keel. Additionally, longitudinally-oriented microgrooves along the keel surface might be adequate to replace such peripheral extensions in addressing rotational stability. A laser-induced microgroove architecture along the TT surface interfacing with cement that can offer sufficient torsional stability to TT in cemented and cementless TKR systems is ready for future study. Since the study goals included developing novel laser micromachining schemes for metallic implants, the experiments were limited to one sample per study group for static tests and two samples per study group for cyclic tests. 

Titanium (Ti)- and cobalt-chromium (Co-Cr)-based alloys are the most commonly used materials for joint replacement implants [22]. We used aluminum instead of Ti and Co-Cr due to the machining limitation in our study. It is assumed that metal composition would likely have a negligible influence on the in vitro performance of cemented LIM-treated implants. Identical LIM architecture may be expected to produce the same mechanical interlock regardless of the metal and it is this mechanical interlock that chiefly governs the mechanical stability of a cemented implant [32].

We employed a precision diamond saw machine to create microgrooves on a straight rod implant surface in a previous study [25]. That method of microgrooving is not suitable for orthopedic implants, such as a TKR tibial tray, that feature a complex shape. Other surface roughening techniques, such as surface cutting, etching, and ion deposition techniques, can create roughness on uniform implant surfaces and materials [33]. However, these techniques are not suitable to produce controlled roughness on non-uniform (e.g., keel surface in tibial tray) and multi-metal implant surfaces. Laser engraving holds much potential for producing high precision, rough, and uniform microgroove topography on complex implants [8,13,34].

## 5. Conclusions

In this study, laser-induced microgrooving enhanced the acute mechanical performance of simulated orthopedic implants. A distinctive topographical difference with increased surface area and roughness was achieved and translated into greater force to failure in tension and compression tests. The LIM surface architecture proposed in this study holds much potential for improving the acute performance and durability of joint replacement devices.

## Figures and Tables

**Table 1:** Material properties used for computational analysis.

Properties	Unit	Bone [20]	Aluminum	Cement (PMMA) [21]
Density	kg/m^3^	1550	4400	1770
Young’s Modulus	N/mm^2^	1 × 10^5^	1.06 × 10^5^	2.27 × 10^3^
Poisson’s Ratio		0.45	0.33	0.46

**Table 2:** Difference of average true strain values of smooth and grooved samples.

No of Cycles	Smooth	Grooved
11,000	0.0034 ± 0.0003 (*n* = 2)	0.0046 ± 0.0008 (*n* = 2)
27,000	0.0045 ± 0.0002 (*n* = 2)	0.0104 (*n* = 1)
